# What is the economic evidence for mHealth? A systematic review of economic evaluations of mHealth solutions

**DOI:** 10.1371/journal.pone.0170581

**Published:** 2017-02-02

**Authors:** Sarah J. Iribarren, Kenrick Cato, Louise Falzon, Patricia W. Stone

**Affiliations:** 1 University of Washington, Department of Biobehavioral Nursing and Health Informatics, School of Nursing, Seattle, Washington, United States of America; 2 Columbia University, School of Nursing, New York, New York, United States of America; 3 Office of Nursing Research, EBP and Innovation, New York-Presbyterian Hospital, New York, New York, United States of America; 4 Center for Behavioral Cardiovascular Health, Department of Medicine, Columbia University Medical Center, New York-Presbyterian Hospital, New York, New York, United States of America; 5 Columbia University, School of Nursing, Center for Health Policy, New York, New York, United States of America; Deakin University, AUSTRALIA

## Abstract

**Background:**

Mobile health (mHealth) is often reputed to be cost-effective or cost-saving. Despite optimism, the strength of the evidence supporting this assertion has been limited. In this systematic review the body of evidence related to economic evaluations of mHealth interventions is assessed and summarized.

**Methods:**

Seven electronic bibliographic databases, grey literature, and relevant references were searched. Eligibility criteria included original articles, comparison of costs and consequences of interventions (one categorized as a primary mHealth intervention or mHealth intervention as a component of other interventions), health and economic outcomes and published in English. Full economic evaluations were appraised using the Consolidated Health Economic Evaluation Reporting Standards (CHEERS) checklist and The PRISMA guidelines were followed.

**Results:**

Searches identified 5902 results, of which 318 were examined at full text, and 39 were included in this review. The 39 studies spanned 19 countries, most of which were conducted in upper and upper-middle income countries (34, 87.2%). Primary mHealth interventions (35, 89.7%), *behavior change communication* type interventions (e.g., improve attendance rates, medication adherence) (27, 69.2%), and short messaging system (SMS) as the mHealth function (e.g., used to send reminders, information, provide support, conduct surveys or collect data) (22, 56.4%) were most frequent; the most frequent disease or condition focuses were outpatient clinic attendance, cardiovascular disease, and diabetes. The average percent of CHEERS checklist items reported was 79.6% (range 47.62–100, STD 14.18) and the top quartile reported 91.3–100%. In 29 studies (74.3%), researchers reported that the mHealth intervention was cost-effective, economically beneficial, or cost saving at base case.

**Conclusions:**

Findings highlight a growing body of economic evidence for mHealth interventions. Although all studies included a comparison of intervention effectiveness of a health-related outcome and reported economic data, many did not report all recommended economic outcome items and were lacking in comprehensive analysis. The identified economic evaluations varied by disease or condition focus, economic outcome measurements, perspectives, and were distributed unevenly geographically, limiting formal meta-analysis. Further research is needed in low and low-middle income countries and to understand the impact of different mHealth types. Following established economic reporting guidelines will improve this body of research.

## Introduction

Globally, mobile phone subscribers have grown from less than 1 billion in 2000 to more than 7 billion in 2015, corresponding to a penetration rate of 97% worldwide [1]. Capitalizing on this widespread use of mobile phones, researchers and implementers have used them as a catalyst for healthcare change to address disparities and inequities in health service access and delivery, geographic barriers, shortage of health care providers, and high health care costs [2, 3]. Mobile health (mHealth) is defined as the “medical and public health practice supported by mobile devices, such as mobile phones, patient monitoring devices, personal digital assistants (PDAs), and other wireless devices” [4]. Ideally, mHealth improves health outcomes by, for example, efficiently and effectively increasing patient knowledge about a disease/condition, providing social support to those undergoing challenging treatment regimens of stigmatizing diseases, enhancing patient-provider communication, or improving communication and coordination across multidisciplinary care teams thereby improving quality of care delivery [5].

In addition to establishing the effectiveness of these interventions it is also crucial to understand their economic impact given the growing recognition across the globe that resources are finite. Economic evaluations can guide policymakers and funders in determining whether evidence supports wider adoption of mHealth interventions [6]. Such evaluations identify and compare alternative interventions, and assess incremental impact on health outcomes and their costs (differences between intervention under study and comparator intervention) [7].

mHealth is often assumed to be or described as cost-effective or cost-saving, yet the strength of the evidence supporting this assertion has been limited [8–10]. Despite optimism, unknown cost-effectiveness has been listed as one of the top six barriers to mHealth implementation [4] and as a key factor in limited mHealth policy investment [11]. There are a number of ways in which mHealth interventions may reduce health care costs including, but not limited to, decreasing transportation costs for patients or healthcare workers, addressing inefficient practices, decreasing time to diagnosis, keeping patients in their home longer versus costly health care facilities, or reducing hospital visits [12]. Prior systematic reviews of economic evidence of technology-based interventions have focused on telehealth/telemedicine, [13, 14] electronic health (eHealth) and/or specific diseases or populations,[15–18] or a combination of telehealth, eHealth, mHealth [10]. Therefore, to our knowledge, no studies focused on understanding the potential economic impact of mHealth interventions broadly. The aim of this review was to summarize and assess the body of evidence related to economic evaluations of mHealth interventions.

## Methods

The methods for reviewing economic evaluations included (1) applying explicit inclusion criteria to select studies; (2) developing a data abstraction form and characteristic categories to record individual study characteristics; (3) evaluating the quality of reporting of each study using the Consolidated Health Economic Evaluation Reporting Standards (CHEERS) statement checklist [19]; and (4) interpreting and summarizing the identified economic evaluations based on study questions. Questions included: What economic evaluation of mHealth interventions evidence is currently available? What types of mHealth interventions have undergone economic evaluations? What economic evaluation methods were used? What patient population, disease, or health outcomes are targeted? In what settings is research being conducted? Is there any evidence of the cost-effectiveness of mHealth interventions? If so, what is the quality of reporting the economic evidence? Of the identified studies, how many report that the intervention economic outcomes were positive (e.g., cost-effective, economically beneficial, or cost saving) at base case? The protocol for this systematic review was registered in PROSPERO (CRD42014014913) (www.crd.york.ac.uk/PROSPERO/). We followed the Preferred Reporting Items for Systematic Reviews and Meta-Analyses (PRISMA) guidelines for this report [20]. The PRISMA checklist is in Supporting Information S1 Checklist.

### Finding and selecting relevant studies

#### Search strategy

Seven electronic bibliographic databases including MEDLINE (Ovid), EMBASE (EMBASE.com), CINAHL, The Cochrane Center Register of Controlled Trials (CENTRAL), NHS Economic Evaluation Database (NHS EED), Database of Abstracts of Reviews of Effects (DARE), and PsycInfo (Ovid) were searched to April, 2016. Subject headings and text words that encompassed the concepts of mobile health and economic evaluations were used. Search strategies were developed and run by an information specialist (LF). The search strategies are provided in Supporting Information S1 Appendix. Clinicaltrials.gov and WHO International Clinical Trials Registry Platform were searched for ongoing trials and sites such as mHealth Evidence, OpenGrey, HIMSS website were searched for grey literature from October—December, 2015. We further searched the cited references and reference lists of included studies (through ISI Web of Science) and relevant systematic reviews by hand to identify additional relevant studies.

#### Inclusion and exclusion criteria

We included original articles published in English that compared the costs and consequences of at least two interventions, one of which was a mHealth intervention. mHealth interventions could be either the primary intervention or a component of an intervention. We excluded studies not meeting mHealth criteria such as telehealth with stationary devices (e.g., desktop videophone, desktop computer, videoconferencing equipment) unless they reported also using mobile devices (e.g., mobile phone or sensors), were web-based only, or were devices for clinical diagnosis (e.g., EEG) that did not report on a health outcome. We excluded reviews or commentaries of economic evaluations. Protocols for planned economic evaluations of mHealth interventions were identified and included in summary of study characteristics but excluded from the full analysis. While we classified and reported the number of partial economic evaluations identified, which measure only costs of an intervention without comparator (e.g., cost accounting of intervention, cost per patient or cost per an event), these provide limited insight [20].

#### Study selection process

We used a web-based database management system, Early Review Organizing Software (EROS) for data management, developed by the Institute for Clinical Effectiveness in Argentina, to facilitate screening, identify and resolve discrepancies, and to produce the flow diagram. Two reviewers (SI, KC) screened the titles and abstracts of the retrieved records independently and excluded obviously irrelevant records. Full text articles were obtained for studies of possible inclusion for further evaluation. Final decisions were based on the consensus of both screeners. A third reviewer (PS) was included when uncertainty regarding eligibility arose. Authors of published research protocols and conference proceedings were contacted for final report, economic data, or estimated date of publication.

### Development of data abstraction form and study characteristic categories

The data extraction form was developed in MS Excel to capture data for 48 study characteristics based on data points of reviews of economic evaluation [13, 15] and study questions. The data points included the type of economic evaluation, mHealth intervention type, mHealth as the primary intervention versus combined with other intervention strategies, target disease/condition, country, economic outcomes, costs, effectiveness and funding.

mHealth applications have been categorized in different ways. For example, the mHealth Compendium outlines five main categories of mHealth types or applications (e.g., behavior change communication, data collection, finance, logistics, and service delivery), [21] while others use six [22] or twelve categories [5]. To code the mHealth intervention type we used five categories of mHealth interventions and provide definitions and examples of what each include in Table 1. We selected the primary mHealth type with the understanding that other application categories may also be applicable. Often interventions integrate two or more types of mHealth applications (e.g., text message, app) to address a health need or health system constraints [22]. Country income level was classified according to the 2015 World Bank 4 ratings categories (Low income (LIC), lower-middle-income (LMIC), upper-middle-income economies (UMIC), upper income country (UIC)) [23].

**Table 1:** mHealth application types and examples.

Type	Definition of application	Examples of activities
Behavior Change Communication (BCC) or Social BCC	Provide health information and behavior change messages directly to clients or the general public and help link people with services. Message content may increase individuals’ knowledge or influence their attitudes and behaviors.	Appointment reminders Support for medication adherence Promote healthy behavior (e.g. smoking cessation) Community mobilization Awareness-raising, education Apps to support self-management
Information systems / Data collection	Increase the speed, reliability, quality, and accuracy of data collected through electronic methods and send to various levels of health system (district, state, national) for quicker analysis compared to paper-based systems.	Collection and reporting of patient health and service provision Electronic health records (EHR) Registries, vital events tracking, surveillance and household surveys
Logistics / Supply management	Help track and manage commodities, prevent stock-outs, and facilitate equipment maintenance. Transmit information from lower-level to higher level health facility.	Ensure medicines and basic supplies are in stock
Service delivery	Support health worker performance related to diagnosis, treatment, disease management and referrals, as well as preventive services. Provide decision support to patients.	Electronic decision support, point of care tools, checklists, diagnostic tools, treatment algorithms Improve communication: provider-provider, provider-patient (notify test results, follow-up visits)
Financial transactions and incentives	Improve access to health services, expedite payments to providers and health services, and reduce cash-based operating costs.	Load/transfer/withdraw money, savings accounts, and insurance Performance-based incentives, vouchers for services (e.g., family planning and antenatal services)
Workforce development and support	Facilitate training and education, provider work planning and scheduling, supportive supervision, and human resource management.	Train and retain health care workers, provide education

Note. Adapted from the Global Health Learning Center mHealth Basics, USAID (2014) and mHealth Compendium (2015)

There are several types of comprehensive economic evaluations methods including: cost minimization, cost-consequence, cost-effectiveness, cost-utility (i.e., a special type of cost-effectiveness analysis) and cost-benefit analysis [7, 24]. Each type of evaluation compares the costs of alternative strategies but vary in how effectiveness is measured [24]. Outcomes of full economic evaluations include estimates of cost and effectiveness, incremental cost effectiveness ratio (ICER), cost per life saved, disability adjusted life year (DALY), quality adjusted life year (QALY), time-savings gained, and measurement and comparisons of healthcare costs (e.g., costs for buying, implementing, running, representative monetary conversion factors, cost of mobile phone access and provision, and healthcare utilization) [8]. Consequences of health interventions can be evaluated using a number of approaches, for example, a single analytical study, a synthesis of studies, mathematical modeling, or a combination can be used to estimate health consequences [19]. Interpretation of results should reflect the constituents represented and is influenced by assumptions and values used to conduct the evaluation [25]. These assumptions include, for example, the perspective, time horizon, data source, and at which ‘threshold’ an intervention may be considered cost-effective in a given country or setting—such as $50,000 per Quality Adjusted Life Year (QALY) or considering the prevalence or severity of the condition studied. Study outcomes reporting intervention at base case as cost-effective, economically beneficial, or cost saving were coded as having a positive costing outcome or not [Y/N].

### Quality of reporting assessment

To optimize the reporting of economic evaluations, a relatively new checklist of 24 items was developed through expert consensus, the Consolidated Health Economic Evaluation Reporting Standards (CHEERS) statement [19]. Items include reporting target population, time horizon, discount rate, source of effectiveness and cost data, and currency, for example. This checklist has been endorsed by the ISPOR Health Economic Evaluations Publication Guidelines Task Force and co-published across 10 health economics and medical journals to improve reporting and, in turn, health and health care decisions. Full economic evaluations were evaluated for quality of reporting using the CHEERS statement by two authors independently (SI, KC) as a measure to assess risk of bias of reporting economic outcomes [19]. Any discrepancies were resolved with a third reviewer (PS). Although there is no standard, universally accepted method of critical appraisal of economic evaluations, there are several points of methodological quality that can be considered [25]. We used the quantity of reported CHEERS items and considerations described by Henrikson et al (2013) to discuss study quality.

### Interpreting and summarizing economic evaluations

Findings were summarized and reported based on our study questions: number of economic evaluation of mHealth interventions; mHealth interventions types; economic evaluation methods; disease/condition focus; country where conducted; reporting quality; and evidence of the positive costing outcomes (e.g., cost-effective, economically beneficial, or cost saving) at base case. Disparate study designs, intervention type, study context, patient population, and types of economic analysis meant that formal meta-analysis was inappropriate. For this reason, we present a descriptive analysis of the studies included. For data points without predefined categories, as described above, we first free texted study characteristics and then recoded by grouping similar themes (e.g., similar disease/condition focus areas, similar costing perspectives). To summarize reporting quality we (1) categorized highest quality studies as the top 25^th^ percentile (reporting 90–100% of the recommended items CHEERS guideline items); (2) reported most items missing from being reported, and (3) discussed quality evaluation based on the CHEERS assessment domains. To gain insight into what mHealth interventions showed promise, studies categorized as reporting positive costing outcome (eg. cost-effective, economically beneficial, or cost saving) at base case were reported by category in study characteristic summary table.

## Results

### Literature search and evaluation for study inclusion

**Fig 1:**
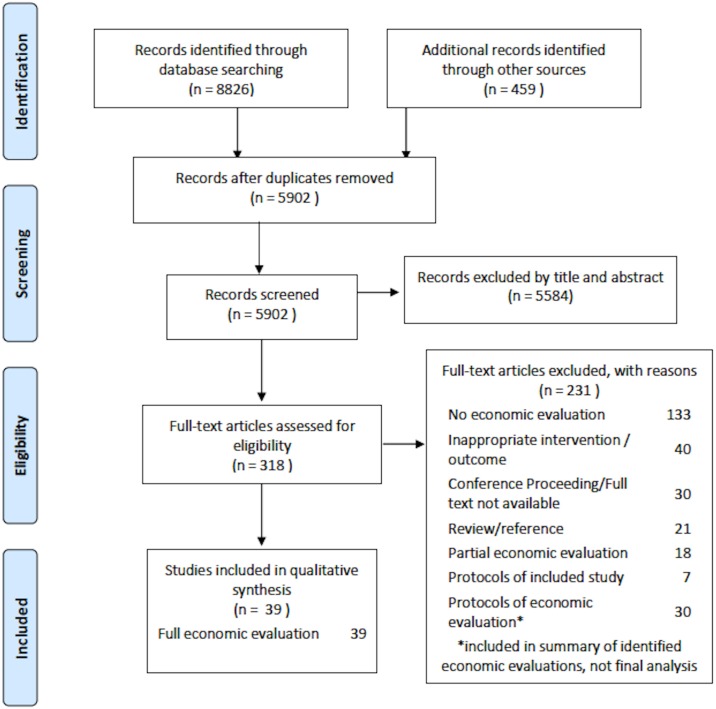
PRISMA flow diagram of study inclusion process.

The searches yielded a total of 8826 results. Subsequent searches in the grey literature and screening through Web of Science resulted in an additional 459. Of the 5902 studies screened after deduplication and excluding those clearly outside inclusion criteria, 318 were screened full text, and 39 economic evaluations were included. Of those excluded from final analysis were 30 protocols with planned economic evaluations and 18 classified as partial economic evaluations. Other reasons for exclusion are described in the PRISMA flow diagram (Fig 1).

### Study characteristics

**Fig 2:**
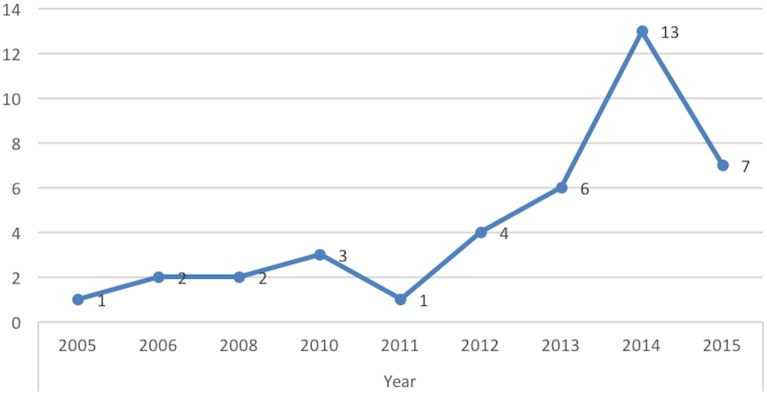
Count of economic evaluation article by year.

The number of published mHealth economic evaluations increased since 2012 (Fig 2). No full economic evaluations were identified up to the 2016 screening date. Of the 30 identified protocols 6 were published in 2013, 8 in 2014 and 16 in 2015. Eight corresponding authors of published protocols reported that their study was in progress and/or expected publication late 2016–2017.

Table 2 provides a summary of the study characteristics and corresponding number of studies with reported costing outcomes categorized as positive (e.g., cost-effective, economically beneficial, or cost saving). Most reported positive costing outcomes at base case (26, 74.3%). The 39 studies spanned 19 countries, the majority of which were conducted in upper and upper-middle income countries (34, 87.2%) compared to 5 in lower-middle or low-income countries (12.8%). Of these 70.6% and 100% reported positive costing outcomes, respectively. The majority evaluated an mHealth intervention as the primary intervention (35, 89.7%) versus as a component of an intervention (4, 10.3%), both categories with about three quarters reporting positive costing outcomes. Twenty seven of the 39 studies used a behavior change communication type interventions (e.g., improve attendance rates, medication adherence) (27, 69.2%) with high rates of reported positive costing outcomes (20, 74.1%). SMS was the mHealth function most often used in the interventions (e.g., used to send reminders, information, provide support, conduct surveys or collect data) (22, 56.4%) with 17 (77.3%) resulting in reported positive economic outcomes. The most frequent disease or condition focuses were outpatient clinic attendance, cardiovascular disease, and diabetes.

**Table 2:** Study characteristics summary of economic evaluations with reported positive costing outcomes.

	n = 39 No(%)	Positive costing outcome within category No(%)
**Country**		
US	9(23.1)	7(77.8)
UK	6(15.4)	4(66.7)
African Countries (Malawi, Kenya, Uganda, Cameroon)	5(12.8)	5(100)
Other European countries (Sweden, Spain, Switzerland)	4(10.3)	4(100)
Other Countries (Canada, New Zealand, Korea, Mexico)	4(10.3)	1(25)
China	3(7.65)	2(66.7)
Australia	3(7.69)	3(100)
Thailand	2(5.13)	1(50.0)
Malaysia	2(5.13)	1(50.0)
Multi-country study (South Africa, Mexico, Guatemala)	1(2.56)	1(100)
**Country by income level**		
Upper income country (UIC)	25(64.10)	19(76.0)
Upper-middle-income economies (UMIC)	9(23.08)	5(55.6)
Lower-middle-income (LMIC)	2(5.13)	2(100)
Low income (LIC)	3(7.69)	3(100)
**mHealth as primary intervention or component in other interventions**		
Primary intervention	35(89.7)	26(74.3)
Component of intervention	4(10.3)	3(75.0)
**mHealth type**		
Behavior change communication	27(69.2)	20(74.1)
Data collection	7(18.0)	4(57.1)
Service delivery	5(12.8)	5(100)
**Intervention focus**		
Outpatient clinic attendance	7(17.95)	6 (85.7)
Cardiovascular diseases (e.g., Heart failure, hypertension)	5(12.8)	4(80.0)
Diabetes	4(10.3)	3(75.0)
Pulmonary (e.g., asthma, COPD, smoking)	3(7.69)	2 (66.7)
Screening, surveillance (e.g., cancer)	3(7.69)	2 (66.7)
HIV/AIDS	2(5.13)	1 (50.0)
Risk assessment/reduction	2(5.13)	1(50.0)
Obesity	2(5.13)	1(50.0)
Tuberculosis	2(5.13)	1(50.0)
Maternal/child care	2(5.13)	1(50.0)
Mosquito born (Dengue, malaria)	2(5.13)	1(50.0)
Decision support	2(5.13)	2(100)
Physical Activity	1(2.56)	1(100)
Post-surgical f/u	1(2.56)	1(100)
Vaccinations	1(2.56)	1(100)
**mHealth related function**		
SMS (e.g., reminder, information, support)	22(56.41)	17(77.3)
Mobile application (App)	9(23.1)	5(55.6)
Multiple (e.g., app and SMS, SMS and IVR/wireless devices)	1(2.56)	1(100)
PDA, palm pilot	1(2.56)	1(100)
Sensors (fall, heart, ingestible), digital devices (smoke detector connected to phone)	3(7.69)	3(100)
SMS survey or data collection	3(7.69)	2(66.7)
**Economic evaluation type**		
CEA	25(64.1)	18(72.0)
CUA	12(30.8)	10(83.3)
CMA	1(2.56)	0(0)
CBA	1(2.56)	1(100)
**Costing perspective**		
Not reported	16(41.0)	12(75.0)
Payer/Health Service Provider/Program/Employer	12(30.77)	9(75.0)
National Health Service (including US military / Civilian)	5(12.8)	3(72.0)
Healthcare System and patient	2(5.13)	2(100)
Multiple (healthcare system, government, patients)	2(5.13)	1(50.0)
Societal and health care system	1(2.56)	1(100)
Societal	1(2.56)	1(100)

Note: SMS = Short message service, CEA = Cost-effectiveness analysis, CUA = Cost utility analysis, CMA = Cost minimization analysis, CBA = Cost benefit analysis

Cost-effectiveness analysis (CEA) was the predominant economic evaluation method (25, 64.1%) and cost utility analysis (CUA) had the highest within group positive costing outcomes (10, 83.3%). The costing perspective was mostly from the payer/health service provider/program/employer perspective; however, a large number did not clearly report the costing perspective (16, 41%).

### Quality of reporting of full economic evaluations (CHEERS)

Supporting Information S2 Appendix provides the evaluation of each study by CHEERS item checklist. Table 3 summarizes the CHEERS items missing when expected for all studies. The average percent of items reported in the studies was 79.6% (range 47.62–100, STD 14.18). The five items most likely not to be reported were: characterization of heterogeneity (29, 74.4%); characterizes uncertainty—sensitivity of incremental costs (single study-based) (17, 61.7%); identifying the study as an economic evaluation in the title (19, 48.7%); stating currency, price date, conversion (18, 46.2%); and stating study perspective (16, 41%).

**Table 3:** CHEERS evaluation criteria summary of missing items.

CHEERS criteria	Number of items missed (Total No count)	Percent of studies missing items
Title Identified Economic	19	48.72
Structured Abstract	2	5.13
Intro Has Context	0	0
Population Characteristics	1	2.56
Setting/ Location	1	2.56
Study Perspective	16	41.03
Comparators Described	0	0
Time Horizon	9	23.08
Discount Rate	3	16.67
Describes Outcome Measures	1	2.63
Measurement of Effectiveness (Single Study Based Estimates)	0	0
Measurement of Effectiveness (Synthesis-Based Estimates)	0	0
Preference Based Outcomes	0	0
Est. Resources and Costs (Singe Study-Based)	0	0
Est Resources and Costs (Model-Based)	0	0
Currency, Price Date, Conversion	18	46.15
Describes Choice of Model	3	15
Describes Assumptions	10	25.64
Describes Analytic Methods	6	15.38
Reports Study Parameters	5	12.82
Reports Incremental Costs and Outcomes	13	34.21
Characterizes Uncertainty—Sensitivity of Incremental Costs (Single Study-Based)	17	60.71
Characterizes Uncertainty—Sensitivity of Incremental Costs (Model-Based)	0	0
Characterizes Heterogeneity	29	74.36
Summarizes Findings, Limitations, Current Knowledge	0	0
Describes Funding Source	5	12.82
Conflict of Interest	14	35.9

Note. Item characterized as missing when expected and not present

Twelve (30.7%) of the full economic evaluations reported greater than 90% of the CHEERS items, ranking them in the 25^th^ percentile, thus are considered of higher reporting quality [26–37]. Supporting Information S3 Appendix provides a summary of these studies intervention comparators, time horizon, discount rate, outcomes and findings. These studies were all published between 2012 and 2016, whereas the publication dates of those not consistent with CHEERS items ranged from 2005 to 2015. Of the economic evaluations reporting the highest CHEERS items, nine (75%) were *behavior change communication* type based interventions using text messaging as the primary intervention to send reminders or support [28–32, 34, 36, 37] or an app [35]. Two were *service delivery* based using an app to replace surgical follow-up visits [27] and an iPhone based sensor for screening [33, 27]. One was primarily for *data collection* using a personal digital assistant (PDA) [26]. There was a range of disease/condition focuses, for example, tuberculosis control,[31] diabetes prevention and management, [26, 36] and malaria management [37]. Most were CUA (8, 66.7%), the remainder were CEA. Of these studies, 4 used primary RCT as their effectiveness data source [28, 30, 34, 37] and 5 drew from prior RCT or multiple studies [27, 29, 31, 32, 36]. Eleven used usual care, current practice or a control group not receiving intervention as the comparator intervention and one used medication self-administration as the comparator group [31]. The interventions and comparators for top 25^th^ percentile reporting are listed in Appendix B and Appendix C summarizes studies by mHealth type. Five applied a lifetime time horizon [29–31, 33, 36] while others used four years or less. The majority applied a discount rate of 3–5% (Add definition of what a discount rate is) (9, 75%) [26, 28–33, 36]. Only two calculated costs based on a societal perspective [27, 30]. All conducted sensitivity analyses.

In contract, in the lowest 25^th^ percentile (n = 11), 5 used RCT data [38–41] while others used effectiveness data from pilot or observational studies [42–47]. However, none reported the study perspective or characterized heterogeneity, and only one [48] included sensitivity analyses. Study duration for all were reported as under 6 months, therefore, not requiring discount rates [48].

### Protocols with planned economic evaluations

Twenty-eight of the protocols of mHealth based interventions describe full economic evaluations (five from the grey literature). Of these, one is a primary CEA, one is a prospective cohort study with planned CEA, another is a fractional factorial design with CEA and the remaining are RCTs with CEA. Two were categorized as partial economic evaluations (e.g., cost accounting, and partial and direct costs). One focuses on *data collection* mHealth intervention type, 7 focus on *service delivery* and the majority focus on *behavior change communication* based interventions (26, 87%). The focus conditions vary widely. For example, six will focus on HIV and four each on diabetes, physical activity, and pulmonary issues. Other topics include risk reduction of binge drinking, self-harm, and injury prevention.

## Discussion

### Overview

Economic evaluations facilitate the comparison between interventions in terms of their costs and consequences and can be used to guide decision makers or funders in determining if mHealth-based interventions improve health outcomes relative to other existing interventions and if the cost to adopt and maintain the intervention in a system or setting is justified [49]. mHealth guidelines recommend the use of economic evaluation tailored reporting standards, such as the CHEERS checklist, for full economic evaluations and support the reporting of basic costs assessment of the mHealth intervention from varying perspectives [50]. In our systematic review, we provide a summary of the economic evidence of mHealth and confirm the common criticism that cost-effectiveness is often assumed, without evidence to support it. In fact, of the excluded studies during screening, 57% included statements of cost-effectiveness or cost in the abstract or title but upon further evaluation did not provide enough detail to be considered a partial or full economic evaluation. However, we did identify more economic evaluations of mHealth than expected given findings from prior reviews [10, 15]. Our review provides an overview of full economic evaluations and highlights a growing number of published planned economic evaluations. Findings show a diverse range of mHealth interventions, focus conditions, and types of mHealth tools used in the interventions evaluated for economic impact. The majority evaluated mHealth as the primary intervention (versus a component of the intervention) and were conducted in upper and upper-middle income countries.

All studies included a comparison of effectiveness of a health-related outcome and reported economic data. However, many did not report all recommended economic outcome items, were not titled/reported as a full economic evaluation, or did not calculate a summary measure. To ensure transparency the authors should provide detail of data sources, assumptions made regarding modeling of data, funding source, and the role of the funder in the analysis and reporting of the study [25]. Over half reported 80% or less of the recommended criteria. Regardless of the quality of reporting, overall there was consistent reporting of positive economic outcomes (e.g., increase in life years gained, cost savings, cost-effectiveness) across mHealth type and cost calculation perspectives. Although findings from this review support cost effectiveness of mHealth interventions, this result must be considered with caution. It is important to evaluate case by case and additional research is needed to identify mHealth components that contribute most to positive outcomes.

Selecting the most appropriate methodology and data collection strategy is important to increase the transferability of findings across economic evaluations [6]. The source of data from randomized control trials or a rigorous prospective cohort study are considered high quality while expert opinion is of low quality due to risk of bias [25]. How outcomes are measured is another quality consideration. High quality outcomes use reliable and validated instruments and/or clinical endpoints (e.g., disease specific) and state how they were calculated and ideally sample people affected by condition or from a general community population [25]. Research designs, sample sizes, and economic reporting quality varied. Three of the studies were pilot study designs,[42, 43, 46] and had small sample size which lack economies of scale (which would indicate the intervention may be even more cost saving) or lack volume (which would indicate more utilization that may increase costs). Some of these studies did not self-report as formal cost-effectiveness studies and instead, for example, reported methods of cost outcome analyses [32, 51]. Additionally, others reported outcomes of effectiveness and difference in costs compared with other interventions or modeled multiple scenarios. Various assumptions about modeling may introduce risk such as using poor quality studies or data that does not reflect current practice that may favor one intervention over the other [25]. For example, Moore et al., assessed effectiveness of a technology supported intervention to support management of hypertension and compared costs between intervention and standard care, identifying cost savings [52]. Similarly, O’Leary et al., evaluated text messaging to increase vaccination outcomes and reported cost scenarios [53]. However, neither calculated a summary measure such as an incremental cost-effectiveness. In another example, Chang et al., calculated costs per outcome averted (e.g., virologic failure and patient lost to follow-up averted) of a peer health worker intervention compared with mHealth supported peer health workers intervention to report patient clinical data to centralized staff. Their findings were based on threshold analyses to identify costs to avert an unwanted outcome and the associated cost savings [54]. Costs and savings were also calculated for implementation at large-scale based on pilot study findings [51]. Efficiency was used as a measure of cost-effectiveness. For example, Bingna et al., defined efficiency as improvements in the primary efficacy outcome relative to the staff working time used and the direct financial costs of the intervention [38]. Cost-effectiveness evaluations were often secondary outcomes or reported in results. Koshy [43] and Perron et al., [40] reported the cost-effectiveness methods within results and discussion sections. Similarly, Loranzo-Fuentes et al., reported cost-effectiveness as a primary study aim, but briefly described the cost analysis methods in the result section [45]. Downer et al., modeled financial benefits that could result from increasing outpatient attendance using text messaging and although the authors report the methods as cost-effectiveness, it was classified as a cost benefit analysis because the outcome was measured as cost per success and the reported cost difference was in monetary units [55]. However, several of the CHEERS quality items were not reported in this study. Joo et al., reported the short intervention period prohibited the calculation of QALYs as a limitation [56].

To be conservative, we reported results as positive or probable cost-effectiveness if one or more of the primary outcomes showed a positive economic outcome. There were cases where secondary outcomes had increased likelihood of cost-effectiveness for an intervention. For example, Maddison et al., identified a mobile phone intervention as not cost-effective compared with usual care for the primary outcome of exercise capacity; however, there were results of cost-effectiveness for secondary outcomes [34]. Similarly, some studies reported the intervention as not cost-effective at base case. However, as the number of patients treated increased, the treatment became less expensive. For example, at approximately 1600 users the app evaluated by Luxton et al., became less expensive than in-office treatment [57]. Furthermore, because of the large number of potential app users, an estimated $USD 2.7–2.9 million societal savings was calculated. The estimates of probability falling below a recognized value threshold for the cost per QALY gained varied based on access to existing software [58].

A main objective of medical research is to improve the health of a population; therefore, conducting economic evaluations from a societal perspective is preferred [59]. The study perspective, such as patient, payer, provider, health systems each take into consideration differing costs and outcomes. A societal perspective is considered high quality because it includes both full direct and indirect costs, such as direct costs to patients and opportunity costs regardless of who bears the costs or receives the effects [25]. Since research is expensive and exposes patients to the risk of experimentation, it is important to think beyond effectiveness of the intervention and to take into consideration the potential to incur opportunity costs [49]. Only two full economic evaluations reported analyses from a societal perspective [27, 30]. Both of these studies also ranked within the top 25^th^ percentile of CHEERS items reported, representing good quality economic evaluations. Those with lower quality score often did not describe the perspective. In such cases from the costs calculated it can often be assumed that the perspective is from the intervention implementer and sometimes the patient or service user costs are also included. Some studies included a mix of perspectives (e.g., provider or health care sector and service user) or at various levels of implementation (e.g., start up, regional level, national level). Similarly, although the majority of the studies included the currency used in their analyses, of those categorized as not reporting the CHEERS item ‘currency, price date, and conversion,’ most failed to include the date or year the costs were calculated. The lack of this information limits reviewers’ ability to convert and compare to other similar studies. In addition, time horizon was not reported in about a quarter of the studies. A time horizon takes into account preferences for future benefits over immediate benefits and applies a discount rate, typically 3 and 5%. A high-quality study will use a time horizon of over ten years [25]. In evaluations of response rate or clinic attendance, it seems appropriate to use a short time horizon. However, for behavior change interventions, e.g., smoking cessation or adherence to medication for a chronic disease, longer time horizons may be necessary.

As technology based interventions and mHealth, in particular, are relatively new it is not feasible to wait for lifetime data to validate cost-effectiveness. Consequently, it is not surprising that many of the identified economic evaluations used modeling techniques to simulate disease projections over a lifetime or long period while incorporating effectiveness and cost evidence. In so doing it is important to calculate and represent uncertainty [60]. For a study to be considered high quality sensitivity analyses are needed to evaluate factors that most influence results and failing to account for the range of adverse events can induce bias [25]. There are a number of methods used to explore uncertainty in economic evaluations (e.g., one-way and multiway sensitivity analyses, threshold analyses, analyses of extremes and probabilistic sensitivity analyses) [49]. For the studies included in this review that applied a model-based economic evaluation, all described the calculation and reported of uncertainty/sensitivity of incremental costs and most used a Monte Carlo simulation for probabilistic sensitivity analysis. In contrast, for single study-based economic evaluations, the uncertainty of sampling together with the impact of methodological assumptions (e.g., discount rate, study perspective) was described in less than half.

*Behavior change communication* interventions were the most represented mHealth intervention type identified in our review. This finding is consistent with others reporting *behavior change communication* interventions as the predominant and most successful of all mHealth interventions [21]. No economic evaluations were found for three of the domains (*human resource management*, *financial transactions and incentives*, and *logistics/supply management*). Similarly, a review of mHealth in low- and middle-income countries by Hall et al., showed no studies were identified as *human resource management* nor *financial transactions and incentives based* interventions [61]. In addition, although mHealth is recognized as drawing from a range of tools and often combined with other strategies,[5] the majority in our study assessed mHealth tools as the primary intervention type and fewer as a secondary or combined component. One exception was Joo et al., who assessed a remote type Internet based intervention with twice weekly SMS prompts for behavior modifications in combination with other intervention components [56].

Text messaging was the predominant tool assessed in the studies, consistent with mHealth literature [21, 62]. Text-messaging interventions are popular because they can be sent, stored, answered and retrieved at the user’s convenience; they are relatively inexpensive; and they are available for any type of phone [61, 63–65]. Increasingly, SMS reminder systems are being used by healthcare systems to counter the negative impacts of missed appointments, such as lost revenue to the health care system, potential poor impact on patient health and treatment outcomes, and system efficiency [66]. The largest number of identified economic evaluation studies focused on assessing cost outcomes of SMS appointment reminders to increase outpatient clinic attendance. All but one found positive economic outcomes [43]. In our review, four focused on diabetes management or prevention, and each of these identified the intervention as cost-effective. However, a systematic review of text messaging interventions concluded that text messaging benefits are still unclear because most studies have used self-reported adherence measures, omitted the measurement of clinical outcomes, and neglected to evaluate beyond the active intervention period to determine lasting effects or to assess baseline adherence difference [65, 67].

Apps are reported to be an ideal platform for behavior change because of their popularity, connectivity, and increased sophistication [68]. Apps can support added functionalities beyond, text messaging, for example. They have the potential for real-time data collection, graphic feedback, interactivity, and links to social functionalities. In our review there were apps identified as cost-effective for follow-up care for low-risk postoperative ambulatory patients [27]. Telephone follow-up may decrease costs and time for follow-up care compared with in-person, but requires synchronous communication and often multiple calls. In contrast, mobile app follow-up can collect and relay data asynchronously and to those in need of evaluating the information, such as surgeons [27].

Interventions to improve disease detection or point of care testing using mobile phone based technologies is an area of mHealth considered to have high potential to increase access to rapid testing and be cost savings [69, 70]. In our study, we identified an app to screen for atrial fibrillation [33]. Authors have noted that widespread implementation remains subject to several challenges and pending issues [70].

### Implications for researchers, health professionals, and policy makers

The increasing focus of economic evaluations of mHealth interventions means there is a need for careful reporting and rigorous evaluation. This review highlights that there is moderate to high quality of economic reporting, but that there is a lack of evaluations in low-middle and low-income counties. And there is significant heterogeneity in terms of settings, costing strategies, and length of follow-up periods, limiting the conclusions to be drawn. The high-quality studies clearly described what was included in the costs and how they were calculated. We encourage the use of the CHEERS checklist for reporting of economic evaluations and for authors to refrain from including statements of cost-effectiveness in their findings in the absence of an economic evaluation.

### Limitations

We systematically searched mHealth research with economic evaluations and included multiple cost outcomes in the developed search criteria. However, as reflected in the CHEERS evaluation, less than half reported economic evaluation in the title and a clear cost outcome was not always identified in the abstract. In abstracts including statements about a cost outcome, we evaluated the full text for inclusion. Nonetheless, economic evaluations not mentioned in the title or abstract may have been missed. Because we found a high percentage of the studies reporting a positive outcome, it is possible that there may be a publication bias and fewer negative findings are being published. Articles not published in English were not included, which is another limitation.

## Conclusions

The body of economic evaluations of mHealth interventions is growing, evidencing researchers’ response to the call as one of mHealth’s major gaps in further implementation and scale up. A number of the studies were rated as reporting high quality evidence and findings suggest high rates of reporting positive costing outcomes using mHealth interventions compared with usual care or other comparators. Although findings from this review support cost effectiveness of mHealth interventions, this result must be considered with caution. It is important to evaluate case by case. All studies compared intervention effectiveness on a health-related outcome and reported economic data, however many did not report all recommended economic outcome items and were lacking in comprehensive analysis. Further attention is needed to follow established economic reporting guidelines to improve the body of evidence. Due to few similarities in the interventions which precluded any quantitative synthesis such as, varied target disease/condition focus, intervention comparators, economic outcomes measures and an uneven geographical distribution, caution is needed in drawing a conclusion of economic evidence of mHealth interventions to date. Further research is needed in low and low-middle income countries to understand the impact of mHealth components that contribute most to positive outcomes. The growing number of planned economic evaluations, along with improved reporting and further targeted synthesis of economic evaluations, will help guide policymakers and funders.

## Supporting information

S1 Checklist PRISMA checklist. (PDF)

S1 Appendix Search strategies. (DOCX)

S2 Appendix Study evaluation by CHEERS item checklist. (DOCX)

S3 Appendix Summary of high quality economic evaluations. (DOCX)
