# Supplementary material for: What is the economic evidence for mHealth? A systematic review of economic evaluations of mHealth solutions
Source: PLoS One. 2017 Feb 2;12(2):e0170581. doi: 10.1371/journal.pone.0170581 (PMC5289471; doi:10.1371/journal.pone.0170581)
Supplement: S1 Appendix — (DOCX) [file pone.0170581.s002.docx]

**S1 Appendix. Search Strategies**

**MEDLINE**

1. exp cellular phone/

2. exp microcomputers/

3. (smart phone$ or smartphone$ or iphone$ or android or windows or blackberry$).tw.

4. ((handheld or portable or cell$ or mobile) adj2 (phone$ or device$)).tw.

5. (personal digital assistant$ or pda$).tw.

6. (ipad$ or tablet$).tw.

7. text messag$.tw.

8. sms.tw.

9. web.tw.

10. electronic mail/

11. email$.tw.

12. blog$.tw.

13. (chat room$ or chatroom$).tw.

14. podcast$.tw.

15. video recording/

16. or/1-15

17. (cost effective or cost effectiveness).tw.

18. health care costs.sh.

19. sensitivity analys:.tw.

20. or/17-19

21. 16 and 20

22. exp animals/ not humans.sh.

23. 21 not 22

**The Cochrane Library**

#1 MeSH descriptor: [Cellular Phone] explode all trees

#2 MeSH descriptor: [Microcomputers] explode all trees

#3 ("smart phone*" or smartphone* or iphone* or android or windows or blackberry*):ti,ab

#4 ((handheld or portable or cell* or mobile) near/2 (phone* or device*)):ti,ab

#5 ("personal digital assistant*" or pda*):ti,ab

#6 (ipad* or tablet*):ti,ab

#7 "text messag*":ti,ab

#8 sms:ti,ab

#9 web:ti,ab

#10 MeSH descriptor: [Electronic Mail] this term only

#11 email*:ti,ab

#12 blog*:ti,ab

#13 ("chat room*" or chatroom*):ti,ab

#14 podcast*:ti,ab

#15 MeSH descriptor: [Video Recording] this term only

#16 #1 or #2 or #3 or #4 or #5 or #6 or #7 or #8 or #9 or #10 or #11 or #12 or #13 or #14 or #15

#17 "cost effectiveness":ti,ab OR “cost effective”:ti,ab

#18 MeSH descriptor: [Health Care Costs] this term only

#19 "sensitivity analys*":ti,ab

#20 #17 or #18 or #19

#21 #16 and #20

**EMBASE**

#21 #16 AND #19 AND [humans]/lim

#20. #16 AND #19

#19. #17 OR #18

#18. 'sensitivity analysis':ab,ti OR 'sensitivity analyses':ab,ti

#17. (cost AND effectiveness:ab,ti ) OR (cost and effective):ab,ti

#16. #1 OR #2 OR #3 OR #4 OR #5 OR #6 OR #7 OR #8 OR #9 OR #10 OR #11 OR #12 OR #13 OR #14 OR #15

#15. 'videorecording'/de

#14. podcast:ab,ti

#13. 'chat room':ab,ti OR 'chat rooms':ab,ti OR chatroom*:ab,ti

#12. blog*:ab,ti

#11. email*:ab,ti

#10. 'e-mail'/de

#9. web:ab,ti

#8. sms:ab,ti

#7. 'text message':ab,ti OR 'text messages':ab,ti OR 'text messaging':ab,ti

#6. ipad*:ab,ti OR tablet*:ab,ti

#5. 'personal digital assistant':ab,ti OR 'personal digital assistants':ab,ti OR pda*:ab,ti

#4. ((handheld OR portable OR cell* OR mobile) NEAR/2 (phone* OR device*)):ab,ti

#3. 'smart phone':ab,ti OR 'smart phones':ab,ti OR smartphone*:ab,ti OR iphone*:ab,ti OR android:ab,ti OR windows:ab,ti OR blackberry*:ab,ti

#2. 'microcomputer'/de

#1. 'mobile phone'/de

**CINAHL**

S1 (MH "Wireless Communications")

S2 (MH "Microcomputers+")

S3 TI smart phone* OR AB smart phone* OR TI smartphone* OR AB smartphone* OR TI iphone* OR AB iphone* OR TI android OR AB android OR TI windows OR AB windows OR TI blackberry* OR AB blackberry*

S4 TI ( ((handheld or portable or cell* or mobile) N2 (phone* or device*)) ) OR AB ( ((handheld or portable or cell* or mobile) N2 (phone* or device*)) )

S5 TI "personal digital assistant*" OR AB "personal digital assistant*" OR TI pda* OR AB pda*

S6 TI ipad* OR AB ipad* OR TI tablet* OR AB tablet*

S7 TI "text messag*" OR AB "text messag*"

S8 TI sms OR AB sms

S9 TI web OR AB web

S10 (MH "Electronic Mail")

S11 TI email* OR AB email* OR TI e-mail* OR AB e-mail*

S12 TI blog* OR AB blog*

S13 TI "chat room*" OR AB "chat room*" OR TI chatroom* OR AB chatroom*

S14 TI podcast* OR AB podcast*

S15 (MH "Videorecording+")

S16 S1 OR S2 OR S3 OR S4 OR S5 OR S6 OR S7 OR S8 OR S9 OR S10 OR S11 OR S12 OR S13 OR S14 OR S15

S17 (MH "Health Care Costs")

S18 TI cost effective OR TI cost effectiveness OR AB cost effective OR AB cost effectiveness OR TI cost-effective OR TI cost-effectiveness OR AB cost-effective OR AB cost-effectiveness

S19 TI "sensitivity analys*" OR AB "sensitivity analys*"

S20 S17 OR S18 OR S19

S21 S16 AND S20

PsycINFO

1. exp mobile devices/

2. microcomputers/

3. (smart phone$ or smartphone$ or iphone$ or android or windows or blackberry$).tw.

4. ((handheld or portable or cell$ or mobile) adj2 (phone$ or device$)).tw.

5. (personal digital assistant$ or pda$).tw.

6. (ipad$ or tablet$).tw.

7. text messag$.tw.

8. sms.tw.

9. web.tw.

10. computer mediated communication/

11. email$.tw.

12. blog$.tw.

13. (chat room$ or chatroom$).tw.

14. podcast$.tw.

15. audiovisual communications media/ or digital video/ or educational audiovisual aids/ or videotapes/

16. or/1-15

17. exp "costs and cost analysis"/

18. (cost effective or cost effectiveness).tw.

19. sensitivity analys:.tw.

20. or/17-19

21. 16 and 20

22. limit 21 to human

**Clinicaltrials.gov**

mhealth AND cost AND completed studies

mobile AND cost AND completed studies

phone AND cost AND completed studies

pda AND cost AND completed studies

text AND cost AND completed studies

txt AND cost AND completed studies

ipad AND cost AND completed studies

**WHO International Clinical Trials Registry Platform**

Mhealth or mobile or phone or pda or text or txt or ipad in Intervention
