# Supplementary material for: What is the economic evidence for mHealth? A systematic review of economic evaluations of mHealth solutions
Source: PLoS One. 2017 Feb 2;12(2):e0170581. doi: 10.1371/journal.pone.0170581 (PMC5289471; doi:10.1371/journal.pone.0170581)
Supplement: S2 Appendix — (DOCX) [file pone.0170581.s003.docx]

**S2 Appendix. Study Evaluation by CHEERS Item Checklist**

| Author | Economic in Title | Struct-ured Abstract | Intro Provides Context | Pop-ulation Features | Setting/ Location | Study Persp-ective | Comparator Described | Time Horizon | Discount Rate | Describe Outcome Measures | Measurement of Effectiveness (Single Study Based Est.) | Measure-ment of Effect-iveness (Synthesis Based Est.) | Preference Based Outc foomes |
| --- | --- | --- | --- | --- | --- | --- | --- | --- | --- | --- | --- | --- | --- |
| Guerriero* | Y | Y | Y | Y | Y | Y | Y | Y | Y | Y | - | Y | Y |
| Hunchangsith* | Y | Y | Y | Y | Y | Y | Y | Y | Y | Y | - | Y | Y |
| Lowres* | Y | Y | Y | Y | Y | Y | Y | Y | Y | Y | - | Y | Y |
| Burn | Y | Y | Y | Y | Y | Y | Y | Y | Y | Y | Y | - | Y |
| Hagberg* | Y | Y | Y | Y | Y | Y | Y | Y | Y | Y | Y | - | Y |
| Wong* | Y | Y | Y | N | Y | Y | Y | Y | Y | Y | - | Y | Y |
| Larson-Cooper* | Y | Y | Y | Y | Y | Y | Y | Y | Y | Y | Y | - | - |
| Ryan* | Y | Y | Y | Y | Y | Y | Y | Y | - | Y | Y | - | - |
| Zurovac* | Y | Y | Y | Y | Y | Y | Y | Y | - | Y | Y | - | - |
| Adepoju* | N | Y | Y | Y | Y | Y | Y | Y | Y | Y | Y | - | - |
| Armstrong* | Y | Y | Y | Y | N | Y | Y | Y | - | Y | Y | - | Y |
| Maddison* | N | Y | Y | Y | Y | Y | Y | Y | Y | Y | Y | - | Y |
| Cano Martin | Y | Y | Y | Y | Y | Y | Y | N | Y | Y | - | Y | Y |
| Gaziano | Y | Y | Y | Y | Y | N | Y | Y | Y | Y | - | Y | Y |
| Vidal | N | Y | Y | Y | Y | Y | Y | Y | - | Y | Y | - | - |
| Snooks | N | Y | Y | Y | Y | Y | Y | Y | Y | Y | - | Y | Y |
| Chang | Y | N | Y | Y | Y | Y | Y | Y | N | Y | Y | - | Y |
| Rashid | Y | Y | Y | Y | Y | Y | Y | N | Y | Y | Y | - | - |
| Aanesen | Y | N | Y | Y | Y | N | Y | Y | Y | Y | N | - | - |
| O'Leary | Y | Y | Y | Y | Y | Y | Y | Y | - | Y | Y | - | - |
| Luxton | Y | Y | Y | Y | Y | Y | Y | Y | - | Y | - | - | - |
| Joo | Y | Y | Y | Y | Y | N | Y | Y | - | Y | Y | - | - |
| Leong | N | Y | Y | Y | Y | Y | Y | Y | Y | Y | Y | - | - |
| Downer | N | Y | Y | Y | Y | Y | Y | Y | - | Y | Y | - | - |
| White | N | Y | Y | Y | Y | Y | Y | N | N | Y | Y | - | - |
| Katalenich | N | Y | Y | Y | Y | N | Y | Y | - | Y | Y | - | Y |
| McInnes | N | Y | Y | Y | Y | Y | Y | N | - | Y | Y | - | - |
| Milne | Y | Y | Y | Y | Y | N | Y | Y | N | Y | Y | - | - |
| Au-Yeung | Y | Y | Y | Y | Y | N | Y | Y | - | - | - | Y | - |
| Nundy | N | Y | Y | Y | Y | N | Y | Y | - | Y | Y | - | - |
| Bigna | N | Y | Y | Y | Y | N | Y | N | - | Y | Y | - | - |
| Chen | N | Y | Y | Y | Y | N | Y | Y | - | Y | Y | - | - |
| Li | N | Y | Y | Y | Y | N | Y | N | - | Y | Y | - | - |
| Moore | N | Y | Y | Y | Y | N | Y | N | - | Y | Y | - | - |
| Koshy | N | Y | Y | Y | Y | N | Y | Y | - | Y | Y | - | - |
| Junod Perron | N | Y | Y | Y | Y | N | Y | N | - | N | Y | - | - |
| Lozano-Fuentes | N | Y | Y | Y | Y | N | Y | N | N | Y | Y | - | - |
| Farley | N | Y | Y | Y | Y | N | Y | Y | - | Y | Y | - | - |
| Mahmud | N | Y | Y | Y | Y | N | Y | Y | - | Y | Y | - | - |
| No. of items missed (Total No. count) | 19 | 2 | 0 | 1 | 1 | 16 | 0 | 9 | 3 | 1 | 0 | 0 | 0 |
| % missing items | 48.72 | 5.13 | 0.00 | 2.56 | 2.56 | 41.03 | 0.00 | 23.08 | 16.67 | 2.63 | 0.00 | 0.00 | 0.00 |

Note. *= top 25^th^ percentile (high quality) economic evaluations; N=item not identified (missing when expected); Y= Item reported; - = not applicable

| Author | Est. Re-sources & Costs (Model-Based) | Currency Price Date, Conv-ersion | Describe Model Choice | Describe Assump-tions | Analytic Methods | Para- meter | Increm-ental Costs & Out-comes | Characterizes Uncertainty^#^  (Single Study-Based) | Characterizes Uncertainty^#^ (Model-Based) | Charact-erizes Heter-ogeneity | Finding Limitations Current Knowledge | Funding Source | Conflict of Interest | Raw Cnt | % Calc |
| --- | --- | --- | --- | --- | --- | --- | --- | --- | --- | --- | --- | --- | --- | --- | --- |
| Guerriero* | Y | Y | Y | Y | Y | Y | Y | Y | - | Y | Y | Y | Y | 24/24 | 100.00 |
| Hunchangsith* | Y | Y | Y | Y | Y | Y | Y | - | Y | Y | Y | Y | Y | 24/24 | 100.00 |
| Lowres* | - | Y | Y | Y | Y | Y | Y | Y | - | Y | Y | Y | Y | 24/24 | 100.00 |
| Burn | Y | Y | Y | Y | Y | Y | Y | - | Y | N | Y | Y | Y | 23/24 | 95.83 |
| Hagberg* | - | Y | Y | Y | Y | Y | Y | Y | - | N | Y | Y | Y | 23/24 | 95.83 |
| Wong* | Y | Y | Y | Y | Y | Y | Y | - | Y | Y | Y | Y | Y | 23/24 | 95.83 |
| Larson-Cooper* | Y | Y | Y | Y | Y | Y | Y | - | Y | N | Y | Y | Y | 22/23 | 95.65 |
| Ryan* | - | Y | - | Y | Y | Y | Y | N | Y | Y | Y | Y | Y | 21/22 | 95.45 |
| Zurovac* | - | Y | - | Y | Y | Y | Y | Y | - | N | Y | Y | Y | 20/21 | 95.24 |
| Adepoju* | - | N | Y | Y | Y | Y | Y | Y | - | Y | Y | Y | Y | 21/23 | 91.30 |
| Armstrong* | - | Y | Y | Y | Y | Y | Y | - | Y | N | Y | Y | Y | 21/23 | 91.30 |
| Maddison* | - | Y | - | Y | Y | Y | Y | Y | - | N | Y | Y | Y | 21/23 | 91.30 |
| Cano Martin | Y | Y | Y | Y | Y | Y | Y | N | - | N | Y | Y | Y | 21/24 | 87.50 |
| Gaziano | Y | Y | Y | Y | Y | Y | Y | - | Y | N | Y | Y | N | 21/24 | 87.50 |
| Vidal | Y | N | N | Y | Y | Y | Y | Y | Y | Y | Y | Y | Y | 20/23 | 86.96 |
| Snooks | - | N | Y | Y | Y | Y | Y | - | Y | Y | Y | N | N | 20/24 | 83.33 |
| Chang | - | Y | Y | Y | Y | Y | N | Y | - | N | Y | Y | N | 19/23 | 82.61 |
| Rashid | - | Y | Y | N | Y | Y | Y | Y | - | N | Y | Y | N | 19/23 | 82.61 |
| Aanesen | - | Y | - | Y | Y | Y | Y | N | - | Y | Y | Y | N | 17/21 | 80.95 |
| O'Leary | - | N | - | Y | Y | Y | N | N | - | N | Y | Y | Y | 17/21 | 80.95 |
| Luxton | Y | Y | Y | Y | Y | N | - | - | Y | N | Y | N | N | 16/20 | 80.00 |
| Joo | - | N | Y | N | Y | Y | N | Y | - | Y | Y | Y | N | 17/22 | 77.27 |
| Leong | - | N | - | N | Y | Y | Y | N | - | N | Y | Y | Y | 17/22 | 77.27 |
| Downer | - | N | - | Y | Y | Y | N | N | - | N | Y | Y | Y | 16/21 | 76.19 |
| White | - | N | Y | Y | Y | Y | Y | Y | Y | N | Y | Y | N | 18/24 | 75.00 |
| Katalenich | - | N | - | Y | Y | Y | N | N | - | N | Y | Y | Y | 16/22 | 72.73 |
| McInnes | Y | Y | N | Y | Y | Y | Y | N | - | N | Y | Y | N | 16/22 | 72.73 |
| Milne | - | Y | - | Y | Y | Y | N | Y | - | N | Y | N | N | 16/22 | 72.73 |
| Au-Yeung | Y | Y | N | Y | Y | N | N | - | Y | N | Y | N | Y | 15/21 | 71.43 |
| Nundy | - | N | - | N | N | Y | Y | N | - | N | Y | Y | Y | 14/20 | 70.00 |
| Bigna | - | N | - | Y | Y | Y | N | N | - | N | Y | Y | Y | 14/21 | 66.67 |
| Chen | - | N | - | N | Y | Y | Y | N | - | N | Y | Y | N | 14/21 | 66.67 |
| Li | - | Y | - | N | Y | Y | N | N | - | N | Y | Y | Y | 14/21 | 66.67 |
| Moore | - | N | - | Y | N | Y | Y | N | - | N | Y | Y | Y | 14/21 | 66.67 |
| Koshy | - | N | - | N | N | Y | N | N | - | N | Y | Y | Y | 13/21 | 61.90 |
| Junod Perron | - | N | - | Y | N | Y | N | N | - | N | Y | Y | Y | 12/21 | 57.14 |
| Lozano-Fuentes | - | N | - | N | Y | N | Y | N | - | N | Y | Y | N | 12/22 | 54.55 |
| Farley | - | N | - | N | N | N | N | N | - | N | Y | Y | N | 11/21 | 52.38 |
| Mahmud | - | N | - | N | N | N | N | N | - | N | Y | N | N | 10/21 | 47.62 |
| No. of items missed | 0 | 18 | 3 | 10 | 6 | 5 | 13 | 17 | 0 | 29 | 0 | 5 | 14 |  |  |
| % missing items | 0.00 | 46.15 | 15.00 | 25.64 | 15.38 | 12.82 | 34.21 | 60.71 | 0.00 | 74.36 | 0.00 | 12.82 | 35.90 |  |  |

Note. *= top 25^th^ percentile (high quality) economic evaluations; N=item not identified (missing when expected); Y= Item reported; - = not applicable, ^#^ Sensitivity of Incremental Costs
