# Supplementary material for: What is the economic evidence for mHealth? A systematic review of economic evaluations of mHealth solutions
Source: PLoS One. 2017 Feb 2;12(2):e0170581. doi: 10.1371/journal.pone.0170581 (PMC5289471; doi:10.1371/journal.pone.0170581)
Supplement: S3 Appendix — (DOCX) [file pone.0170581.s004.docx]

**S3 Appendix. Summary of top 25^th^ percentile Economic Evaluations in order of CHEERS item checklist reporting, from highest to lowest**

| Author (year) Country | Analytic method/ model | Interventions studied/N per group | - Time horizon - Discount rate - Currency   (base year) | Costs Perspective | Outcome | Consequences | Cost range of intervention | Conclusions |
| --- | --- | --- | --- | --- | --- | --- | --- | --- |
| Guerriero (2013) UK | CUA / Markov model | 1. **SMS-based support** 2. control arm (SP=1000) | - Lifetime - 3.5% - UK pounds   (2009-10) | National health service | Development of smoking related disease | QALY gained | £16,120 per 1000 enrolled smokers | Personalized smoking cessation advice and support was cost-effective (£278 per quitter, ICER £141 per LY gained and £89 per QALY gained) |
| Hunchangsith (2012) Thailand | CUA / Monte Carlo simulation | 1. DOT strategies, **SMS reminder** 2. SAT (SP=2000) | - Lifetime - 3% - (I$)Interna-tional dollar   (2005) | Health-care | Tuberculosis related morbidity and mortality | DALY averted, cost of each intervention | I$1100-I$9000 per DALY | The mobile phone reminder system was not cost-effective, because the mortality rate associated with it was much higher than that associated with the other treatment strategies |
| Lowres (2014), Australia | CUA | 1. **iECG (sensor)** screening at 5 treatment adherence levels (40%-80%) (N=1,000) 2. non-screening | - Lifetime - 5% - $AUD - NR | Australian Health Funder | Prevention of stroke | QALY gained | The cost per iECG screen $USD13.5. Base case $AUD 8,509-$AUD 3,888) | ICER: $USD 4,066 per QALY gained and $USD 20,695 for prevention one stroke. Screening with iECG in pharmacies with an automated algorithm was cost-effective and improved with increased treatment adherence. |
| Burn (2015) Australia | CUA | 1. **MobileMums** (n=133) 2. usual care (n=130) | - 2 years - 5% - $AUD   (2014) | Government department | Increased physical activity | Incremental cost per QALY | 8608 AUD per QALY gained | ICER of 8608 AUD per QALY is expected to be cost-effective at a cost-effective threshold of 64,000 AUD. Varying modelling assumptions had little effect on result |
| Hagberg (2014) Sweden | CUA | 1. Individual counseling, home follow-up,**SMS support** (n=31) 2. usual care (n=35) | - Lifetime - 3% - Swedish to USD   (2012) | Societal | Weight loss | QALY gained | $583.80 | Cost per gained QALY $USD 8,643-9,758 (for follow-up year) |
| Wong (2015) Hong Kong | CUA / Markov Model | 1. **SMS program** 2. usual care | - Lifetime - 3% - USD   (2011) | Health service provider | Prevention of diabetes type 2 in prediabetics | LY gained, QALY gained | Net intervention costs $42.03 per subject | SMS intervention gained an additional 0.071 QALY and saved $1020.35 per person |
| Larson-Cooper (2016) Malawi | CEA | 1. **Mobile phone based tips and reminders**, **interactive voice response** 2. Nonusers (9,798 users) | - 1/2011- 6/2013 - 3% - USD   (2013) | Program-matic | Improvement of maternal, newborn, and child health indicators | Cost per user, per contact with user | $29.33 per user and $4.33 per successful contact. | The average cost for each user experiencing a change in indicator ranged from $67 to $355 The sensitivity analyses showed that cost per user could be reduced by 48% if the service were to operate at full capacity. |
| Ryan (2012), England | CEA | 1. twice daily recording and **mobile phone app based transmission of symptoms** (n=145) 2. usual care (n=143) | - 2008-9 - Pounds - year NR | National health service | Asthma control | self-efficacy | Mean difference in cost of mHealth intervention compared with paper-based was minimal (−£1.26, −£51.47 to £48.95) | Mobile technology did not improve asthma control or increase self-efficacy compared with paper based monitoring and was not cost effective. |
| Zurovac (2012), Kenya | CEA | implementation scenarios for **SMS reminders**   1. Trial conditions, 2. by Ministry of Health in same study location 3. National scale | - 1 year - NA - USD   (2010) | Program implementer | Adherence to malaria management guidelines | Cost per child correctly managed | Cost per additional child correctly managed: USD 0.50 under study conditions, 0.36 if implemented by the MoH in the same area, and 0.03 if implemented nationally | Simple text messaging inexpensive under study conditions with substantial economies if deployed at national scale |
| Adepoju (2014) USA | CEA | 1. **Personal digital assistant** (n=81) 2. Chronic diabetes self-management program (n=101) 3. Combined (n=99) 4. usual care (n=95) | - 2 years - 3% - NR | Employer | Diabetes related premature mortality | Total productivity loss (indirect costs only) | $9814 - $11,459 USD | No evidence found that the chronic disease self-management programs examined in the trial affected indirect productivity losses. |
| Armstrong (2014), Canada | CUA / Monte Carlo simulation | 1. **Mobile app** follow-up 2. in-person follow-up visits (SP=1000) | - 30 days - NA | Societal and healthcare system | Ambulatory surgical follow-up | Patient lost leisure time (use varied mobile app success rates (90-96%) 0.04 QALY between no complication and minor skin infection) | mean societal cost of mobile app follow-up care was 135 CAD and in-person was 383.55 CAD per patient | Mobile app follow-up care is suitably targeted to low-risk postop ambulatory patients. It can be cost-effective from a societal and health care system perspective |
| Maddison (2015), New Zealand | CUA | 1. **Personalize SMS package** and secure website with video messages (n=85) 2. usual care (n=86) | - 4 years - 3.5%) - $NZ   (2012) | Public health and clinical | Cardiovascular disease related change in peak O2 from baseline | QALY for outcomes with a significant treatment effect | cost per participant $22.37 | Not effective at increasing exercise capacity (primary outcome) over and above usual care, was effective and probably cost-effective for increasing physical activity |

Note. Disability-adjusted life years (DALYs), Quality Adjusted Life Years (QALY), Life year (LY), DOT=directly observed treatment, SAT=self administration of therapy, SP=simulated population, I$= hypothetical currency representing purchasing power of local currency, ICER=incremental cost-effectiveness ratio, NR=Not reported, NA=Not applicable
